# Delayed Treatment of Diagnosed Pulmonary Tuberculosis in Taiwan

**DOI:** 10.1186/1471-2458-8-236

**Published:** 2008-07-13

**Authors:** Jimmy PS Chern, Duan-Rung Chen, Tzai-Hung Wen

**Affiliations:** 1 Department of Family Medicine, Tao-Yuan General Hospital, Taiwan; 2 Institute of Health Care Organization Administration, College of Public Health, National Taiwan University, Taiwan; 3 Institute of Epidemiology, College of Public Health, National Taiwan University, Taipei, Taiwan; 4 Associate Professor, Graduate Institute of Health Care Organization Administration, College of Public Health, National Taiwan University, Taiwan; 5 R636, Building of Public Health, No.17 Xu-Zhou Road, Taipei, 10020 Taiwan

## Abstract

**Background:**

Mycobacterium tuberculosis infection is an ongoing public health problem in Taiwan. The National Tuberculosis Registry Campaign, a case management system, was implemented in 1997. This study examined this monitoring system to identify and characterize delayed treatment of TB patients.

**Methods:**

Records of all tuberculosis cases treated in Taiwan from 2002 through 2005 were obtained from the National Tuberculosis Registry Campaign. Initiation of treatment more than 7 days after diagnosis was considered a long treatment delay.

**Results:**

The study included 31,937 patients. The mean day of delayed treatment was 3.6 days. Most patients were treated immediately after diagnosis. The relationship between number of TB patients and days of delayed treatment after diagnosis exhibited a Power-law distribution. The long tail of the power-law distribution indicated that an extreme number occur cannot be neglected. Tuberculosis patients treated after an unusually long delay require close observation and follow up.

**Conclusion:**

This study found that TB control is generally acceptabl in Taiwan; however, delayed treatment increases the risk of transmission. Improving the protocol for managing confirmed TB cases can minimize disease transmission.

## Background

Mycobacterium tuberculosis infection has long been a public health problem in Taiwan. The annual incidence of tuberculosis in Taiwan was 71.1/100,000 and 74.6/100,000 in 1997 and 2002, respectively [1]. In aboriginal mountainous areas, the reported incidence is even higher: 243.6/100,000 in 1997. In a geographic analysis, Yeh *et al*. reported that the incidence of TB cases in aboriginal populations in mountain areas decreased with distance from foci in mountain areas. The Yeh study suggested that recent or new infections, not reactivation, explained the high incidence of TB in the general population of Taiwan [2].

A Netherlands study estimated that the average patient with untreated smear-positive pulmonary TB infects more than ten patients annually during the natural course of the disease [3]. However, identifying smear-positive TB cases through control programs and treatment with effective drug regimens can reduce the spread of infections. Therefore, timely and accurate diagnosis of TB and treatment are vital. Delayed treatment can cause more infections per case [4]. Delays can be categorized as patient delays or health care system delays. Identifying when delays occur and the factors related to types of delay can help tuberculosis control programs and medical providers improve diagnosis and treatment efforts [5].

In Taiwan, an aggressive case monitoring system has been in place since 1997 through the efforts of local public health administrations in cooperation with the Taiwan Centre for Disease Control (CDC). This monitoring system requires medical personnel to report all suspected and confirmed cases of TB to city or county local health bureaus. Reliability of the reporting system is ensured by two policies: the no-report-no-reimbursement policy and the notification-fee policy [6]. One policy stipulates that a medical facility failing to report a suspected case cannot receive reimbursement by the Taiwan national health care system. The second policy financially rewards the medical facility for reporting suspected cases to local health administrations. This monitoring system collects data as of the dates the TB patient is diagnosed and treated. It provides a unique opportunity to study delayed treatment, defined as the length of time between initial diagnosis and initial treatment. Therefore, this study identified and characterized TB cases with unusually long delays in treatment to evaluate the effectiveness of TB control in Taiwan.

## Methods

### Data source

Data for all tuberculosis cases treated during 2002–2005 were obtained from the National Tuberculosis Registry Campaign surveillance program. The TB surveillance program was established by the Center for Disease Control (CDC, Taiwan), Taiwan Department of Health, and began collecting demographic, geographic, diagnostic and treatment data for all diagnosed TB cases since 1997. This study enrolled only TB patients with sputum smear- and/or culture-positive TB. Treatment delays were measured as the time from the date of definite diagnosis (*i.e*., confirmation by laboratory findings) to the date of initial treatment for the disease. Any treatment initiated more than seven days after diagnosis was considered delayed treatment.

In Taiwan, physicians are required to immediately treat patients with confirmed TB. Physicians who lack sufficient expertise in treating TB must immediately refer the patient to a pulmonologist. This referral process is usually accomplished within a week. Data for patients who experienced treatment delays longer than 180 days were considered incorrectly managed and excluded from analysis.

### Statistical analysis

The annual distributions in days of delayed treatment were analyzed. Differences between patients with TB infection were tested by independent *t *test and Chi square test. We employed Mantel-Haenszel Chi-square test for linear trend to examine whether the confirmed TB cases by laboratory diagnosis increased significantly over the four-year period of analysis [7]. A P-value less than 0.05 was considered statistically significant. Distribution-fitting and power function regression were performed to examine whether the days of delayed treatment was Power-law distributed, which was determined by the following formula:

(1) *P*(*k*) ~ *k*^-*γ *^

where *k *represents the total days of treatment delay, and P(*k*) indicates the number of TB cases receiving delayed treatment. If the days of delayed treatment could be fitted by a Power-law function with a high *γ *value (larger than 2), the number of TB cases would sharply decrease, indicating that long delays are rare [8]. Most patients have experienced short delay, but a significant number of nodes have experienced a longer delayed treatment. The LOTKA was used to fit the Power-law distribution [9]

Multivariate logistic regression was employed to model the variables associated with long treatment delay. The dependent variable is a binary variable. Patients with treatment initiated more than seven days after diagnosis was considered delayed treatment, coded as "1", otherwise, coded as "0".

## Results

### Study population

The study analyzed 31,937 patients (4,432 in 2002, 7,146 in 2003, 9905 in 2004, and 10454 in 2005). The incidence of cases confirmed by laboratory diagnosis increased significantly over the four-year period of analysis (Table 1). More males (22,294 males vs. 9,643 females) than females had pulmonary TB infections. The mean age at diagnosis was 66.5 y/o for males vs. 61.7 y/o for females (P < 0.05). Most patients with definite laboratory diagnosis were treated in hospitals (95.6%). The average days of delayed treatment (3.4 days) revealed no gender difference (Table 2).

**Table 1:** The incidence of TB patients with definite laboratory diagnosis in Taiwan during 2002–2005

	TB cases with definite laboratory diagnosis	Total Population	Incidence of TB cases with definite laboratory diagnosis (/100,000 population)
2002	4432	22520,776	19.7
2003	7145	22604,550	31.6
2004	9905	22689,122	43.7
2005	10454	22770,383	45.9

The incidence of cases with definite diagnosis increased significantly during these years. Mantel-Haenszel Chi-square for linear trend p < 0.001.

**Table 2:** Characteristics of TB patients with definite laboratory diagnosis in Taiwan during 2002–2005

Years	2002	2003	2004	2005	Total
Patients (n)	4432	7146	9905	10454	31937
Sex (M/F ratio)	2.32	2.38	2.3	2.27	2.31
Age*	66.3 ± 19.1	65.1 ± 18.7	65.5 ± 19.4	64.7 ± 19.2	65.2 ± 19.2
Male	67.9 ± 18.0	66.7 ± 18.0	66.4 ± 17.8	65.9 ± 18.1	66.5 ± 18
Female	62.5 ± 21.6	61.8 ± 21.8	62.4 ± 22.0	60.8 ± 20.8	61.7 ± 21.3
Patients with known treatment facilities	3430 (77.4%)	5505 (77.0%)	7742 (78.2%)	8305 (79.4%)	24982 (78.2%)
Hospital	3256 (94.5%)	5235 (95.1%)	7406 (95.7%)	7984 (96.1%)	23881 (95.6%)
Primary-care clinic	174 (5.1%)	270 (4.9%)	336 (4.3%)	321 (3.9%)	1101 (4.4%)
Delay (days)	6.1 ± 17.3	3.3 ± 12.1	3.1 ± 11.4	3.0 ± 11.1	3.4 ± 12.3
Male	6.2 ± 17.6	3.4 ± 12.2	3.2 ± 11.2	3.2 ± 11.0	3.5 ± 12.1
Female	4.9 ± 13.9	3.0 ± 9.8	3.0 ± 10.4	2.8 ± 11.0	3.2 ± 11.2

Data are *n*, means ± SD, or *n *(%). Number (*n*) of patients is given when the variable concerned is not measured in all patients.

* Chi-square tests *P *< 0.05.

### Distribution of treatment delays

The distribution of treatment delays was skewed. Mean treatment delay was 6.1 days in 2002, 3.3 days in 2003, 3.1 days in 2004 and 3.0 days in 2005. Median and mode of treatment delay was 0 during these years. In more than 95% of the patients, treatment commenced within 10 days after laboratory-confirmed diagnosis; only 0.5%–1% had treatment delays longer than 100 days (Table 3).

**Table 3:** Distribution of the treatment delay for TB patients with definite laboratory diagnosis in Taiwan during 2002–2005

	2002		2003		2004		2005	
Delay (Day)	N	%	N	%	N	%	N	%

0	2449	55.3	3993	55.9	5331	53.8	5703	54.6
1~10	1402	31.6	2720	38.1	4026	40.6	4215	40.3
11~20	279	6.3	201	2.8	270	2.7	250	2.4
21~30	114	2.6	77	1.1	99	1.0	106	1.0
31~40	46	1.0	52	0.7	49	0.5	47	0.4
41~50	31	0.7	20	0.3	30	0.3	31	0.3
51~60	21	0.5	21	0.3	18	0.2	19	0.2
61~70	21	0.5	10	0.1	24	0.2	14	0.1
71~80	18	0.4	9	0.1	13	0.1	9	0.1
81~90	11	0.2	7	0.1	5	0.1	10	0.1
91~100	8	0.2	12	0.2	8	0.1	6	0.1
101~180	32	0.7	24	0.2	32	0.4	44	0.3
Total	4432	100.0	7146	100.0	9905	100.0	10454	100.0
Mean of Treatment delay	6.1 days		3.3 days		3.1 days		3.0 days	
Median/Mode of treatment delay	0.0 days		0.0 days		0.0 days		0.0 days	

### Patients with long treatment delay

Delayed treatment, defined as initial treatment seven or more days after diagnosis, was noted in 2,813 patients (8.8%). The total number of patients experiencing treatment delays were 743 (16.8%) in 2002, 598 (8.4%) in 2003, 757 (7.6%) in 2004 and 715 (6.8%) in 2005. The percentage of patients experiencing long delays decreased significantly between 2002 and 2005. More males than females experienced long treatment delays (M/F ratio: 2.52). In patients with long treatment delays, males were older than females (67.6 years versus 63.2 years, respectively, p < 0.05), and males tended to have longer delays than females (28.8 days versus 26.4 days, respectively, p < 0.05) (Table 4).

**Table 4:** Characteristics of TB patients with long treatment delay in Taiwan during 2002–2005

Year	2002	2003	2004	2005	Total
Patients (n, %)	743 (16.8%)	598 (8.4%)	757 (7.6%)	715 (6.8%)	2813 (8.8%)
Sex (M/F ratio)	2.52	2.58	2.41	2.58	2.52
Age*	68.1 ± 18.4	67.5 ± 18.1	66.3 ± 18.6	64.1 ± 19.1	66.2 ± 18.4
Male	69.3 ± 17.2	68.3 ± 17.3	67.7 ± 16.9	65.2 ± 18.9	67.6 ± 17.3
Female	63.7 ± 20.1	65.3 ± 21.2	62.5 ± 21.3	61.6 ± 20.0	63.2 ± 20.5
Delay (days)**	29.2 ± 31.5	27.3 ± 30.4	27.1 ± 30.6	29.7 ± 32.1	28.0 ± 31.1
Male	30.9 ± 32.5	28.1 ± 31.1	27.6 ± 30.7	28.4 ± 31.3	28.8 ± 31.3
Female	24.6 ± 27.2	25.1 ± 25.4	25.9 ± 29.4	29.7 ± 33.8	26.4 ± 29.2
Patients with known treatment facilities	568 (76.4%)	429 (71.7%)	589 (77.8%)	518 (72.4%)	2104 (74.8%)
Hospital	542 (95.4%)	411 (95.8%)	565 (95.9%)	505 (97.5%)	2023 (96.2%)
Primary-care clinic	26 (4.6%)	18 (4.2%)	24 (4.1%)	13 (2.5%)	81 (3.8%)

Data are *n*, means ± SD, or *n *(%). Number (*n*) of patients is given when the variable concerned is not measured in all patients.

* Chi-square test, *P *< 0.05.

** t-test, *P *< 0.05.

**Figure 1:**
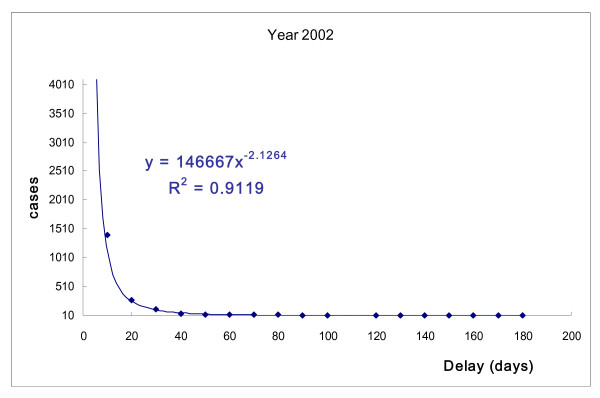
**Number of TB patients and days of treatment delay (2002).** Power-law distribution test, *P *< 0.001.

The correlations between number of TB patients and days of delayed treatment after diagnosis were similar for each year (Fig. 1 to Fig. 4). The days of delayed treatment of TB patients exhibited a Power-law distribution with a 95% statistical significance, indicating that most patients were treated immediately after diagnosis. Conversely, the absolute values of *γ *in power-functions ranged from 2.12 to 2.35 with R-square = 0.90 (Fig. 1 to Fig. 4). This finding suggests that a small number of patients experienced treatment delays for a significant number of days.

**Figure 2:**
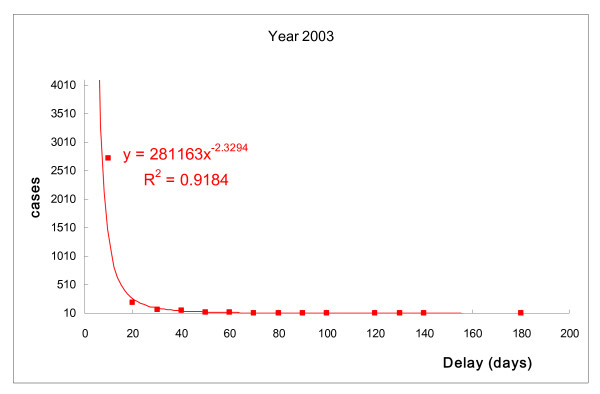
**Number of TB patients and days of treatment delay (2003).** Power-law distribution test, *P *< 0.001.

**Figure 3:**
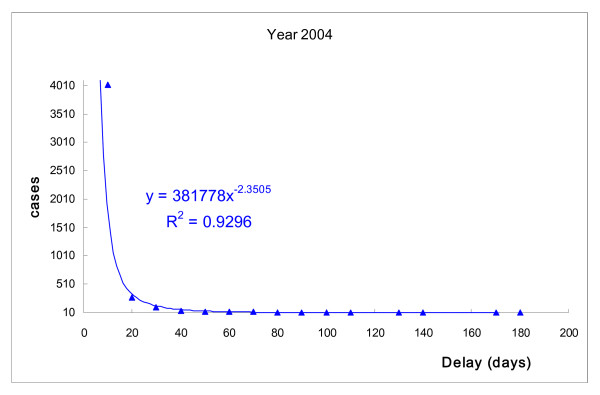
**Number of TB patients and days of treatment delay (2004).** Power-law distribution test, *P *< 0.001.

**Figure 4:**
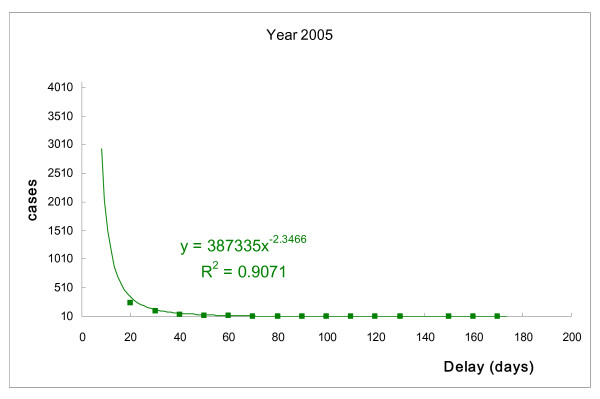
**Number of TB patients and days of treatment delay (2005).** Power-law distribution test, *P *< 0.001.

Multivariate logistic regression results showed that gender, type of treatment facilities, and year were significantly associated with being long treatment delay. As compared to women, men were 1.23 times more likely to be in long treatment delay. Patients treated in hospitals were also 1.28 times more likely to be in long treatment delay, compared to patients treated in clinics. Yearly difference was also significant. The prevalence of long treatment delay in 2003 was 50% less than that in 2002 (the baseline), and the prevalence of long treatment delay in 2004 and 2005 were 57% and 61% respectively less than that in 2002 (as shown in Table 5).

**Table 5:** Logistic regression on long treatment delay

Categorical Predictor	Odds Ratio (OR)	95% CI for OR	LR Test
Gender			P < 0.05
Male vs. Female	1.23	1.04–1.45	
Age			ns
Group 1 vs. Group 3	0.48	0.15–1.51	
Group 2 vs. Group 3	1.01	0.87–1.17	
Treatment Facilities			P < 0.01
Hospital vs. Clinic	1.28	1.09–1.50	
Year			P < 0.01
2003 vs. 2002	0.50	0.41–0.62	
2004 vs. 2002	0.43	0.35–0.53	
2005 vs. 2002	0.39	0.32–0.48	

Note: Age group 1 represents young people less than 20 y/o, group 2 adults between 21–64 y/o, and group 3 the aged over 65 y/o.

## Discussion

Delayed diagnosis and treatment of active tuberculosis can be categorized as patient delay or health care system delay. In the health care system, most delays are caused by the diagnosing facility. Such delays can be further categorized as delayed diagnosis or delayed treatment [10]. Regardless of cause, delayed diagnosis and treatment can be catastrophic to those exposed to infected patients [11], particularly medical personnel [12]. For example, the large 2003 TB outbreak involving sixty healthcare workers in a Taipei hospital was attributed to delayed diagnosis and treatment [13].

Minimizing delays in diagnosis or treatment can substantially improve TB prevention. Previous studies measured patient and health care system delay [4,14-16] whereas the current study focused on treatment delays after definite diagnosis in a health care system. The intent was to focus on the severity of delays occurring. In Taiwan, the incidence of laboratory-confirmed diagnoses increased gradually during these years. However, the incidence of TB remained relatively stable. The increased incidence of definite diagnosis may be attributable to improved accuracy of diagnosis by healthcare facilities.

Among these patients, most cases of TB infection in Taiwan were males, which is consistent with previous studies. This gender difference may partly reflect epidemiological differences, including differences in exposure, infection risk, progression from infection to disease, socio-economic status, cultural factors and quality of health care received [1,17-22].

However, although a gender difference in incidence was noted, no gender difference was noted in number of days of delay after definite diagnosis. Taiwan National Health Insurance was implemented in 1995 to provide universal health coverage. The national health insurance program provides accessibility to health care at reasonable cost [23]. This might have increased utilization of medical care by both genders. Additionally, Taiwan also implemented a no-report-no-reimbursement policy in 1997 which penalizes medical facilities for not reporting possible TB cases by denying reimbursement. Together, these measures may have helped improve the surveillance, diagnosis and time to treatment of TB in the overall population.

However, this study revealed that more men than women experienced long delays in treatment. A study in Yemen demonstrated that women were more likely than men to complete tuberculosis treatment [24], which suggests that women have shorter treatment delays than males and tend to receive treatment immediately after diagnosis. However, a Bangladesh study showed that women experience longer total delay, total diagnostic delay, patient delay and treatment delay (males1.9 days, females 2.0 days) [25]. These inconsistencies highlight the impact of different communities and cultures on gender differences in tuberculosis treatment delays. Hence, a better understanding of the people and communities affected by tuberculosis is needed to provide consistent and high quality care [26].

Recent research indicates that many natural and social-economic phenomena, such as income, disease-related death and earthquake magnitude, follow a Power-law distribution rather than a bell-curve distribution. This implies that small occurrences are common, and large instances are rare but possibly devastating [27,28]. Our study also revealed a Power-law distribution in TB treatment, suggesting that while most TB cases are controlled by public health authorities, the few patients who experience long delays in treatment can cause serious transmission. As can be seen in Fig. 1 to Fig. 2, the tail of the power-law distribution has a much slower decay than other probability distributions, such as the Poisson distribution. The probability that an extreme number will occur from a random sample cannot be neglected if we are dealing with power-law phenomena. It is worth noting that a Power-law distribution in TB treatment, suggesting that while most TB cases are controlled by public health authorities, the few patients who experience long delays in treatment can cause serious risk for transmission [29,30]. As Pastor-Satorras and Vespignani [29] has revealed, in a power-law distribution, epidemics can reproduce with a considerably lower number of infected persons at each point in time, than other probability distributions. Therefore, epidemics in a power-law distribution will not exhibit a threshold. It brings serious concerns for public health researchers working in the field of tuberculosis control.

Additionally, based on the data in this study, long delays in treatment usually occurred in hospitals (range: 94.5% ~ 96.1%) rather than in primary-care clinics. The likely explanation is that not all physicians are familiar with treatment of TB, especially in hospitals with many specialists. Chung *et al*. reported that physicians who are not pulmonologists are less effective in treating TB [31]. We speculate that if the attending physician cannot explain the disease as convincingly as a pulmonologist, the patient may choose to visit another doctor for a second opinion or simply leave under the impression that the doctor is uncertain or is reluctant to disclose the TB findings because of the social stigma attached to the disease. To minimize treatment delays and disease transmission, the protocols for controlling TB at the hospital level must be strengthened. For example, nurses responsible for hospital TB control should be informed immediately after a laboratory diagnosis is confirmed. The nurse can contact the patient, inform primary doctor and arrange an immediate visit to a pulmonologist.

Nevertheless, this study bears the following limitations. First, the data analysis did not differentiate newly detected cases from relapses. Second, global surveillance of drug resistance has shown that a substantial proportion of tuberculosis patients are infected with drug resistant Mycobacterium tuberculosis strains [32]. Unfortunately, the possibility of multiple-drug resistant TB (MDR-TB) cannot be considered due to the lack of information in the data set. However, based on the report from Chest hospital in Taiwan, multidrug resistance occurred in 42.2% of retreated TB patients, and 1.8% of multidrug resistant isolates were found in new TB patients from January 2002 to June 2004 [33]. A recent report in May 2008 revealed that, among 215 patients with MDR-TB, 42.8% (92/215) were fluoroquinolone-resistant [34]. Fluoroquinolones are widely used for the treatment of bacterial respiratory infections in Taiwan. This treatment regimen of using fluoroquinolone before definite diagnosis of pulmonary TB might cause temporary symptom relief of the patient, and might eventually cause treatment delay and drug resistance [34]. Further studies are needed to examine the extent of fluoroquinolone-resistance in patients with long treatment delays.

## Conclusion

This study found that TB control is generally acceptable in Taiwan; however, delayed treatment increases the risk of transmission. Improving the protocol for managing confirmed TB cases can minimize disease transmission.

## Pre-publication history

The pre-publication history for this paper can be accessed here:


